# Posterior element lumbar osteochondroma presenting with severe radiculopathy: A case report

**DOI:** 10.1016/j.radcr.2026.05.018

**Published:** 2026-06-03

**Authors:** Yohannes Girma Zewdie, Biniyam Beyene Tabor, Hana Yeshewas Genetirune, Ermias Fikru Yesuf

**Affiliations:** a Radiologist Department of Radiology, Addis Ababa University, Addis Ababa, Ethiopia; b Radiologist Department of Radiology, Saint Paul millennium medical college, Addis Ababa, Ethiopia; c Department of Anesthesia, Addis Ababa University, Addis Ababa, Ethiopia; d Neurosurgeon Department of Surgery, Debre Berhan University, Debre Berhan, Ethiopia

**Keywords:** Lumbar spinal osteochondroma, Posterior element, Radiculopathy, Spinal canal stenosis, Cortical and medullary continuity, Surgical excision

## Abstract

Spinal osteochondroma is a rare benign tumor that may lead to neurological symptoms when it encroaches on neural structures. We report a 39-year-old male presenting with progressive left lower limb radicular pain for over 3 months, refractory to conservative management. Imaging revealed a bony outgrowth arising from the inferior articular process of the L3 vertebra with cortical and medullary continuity, extending into the spinal canal and causing severe stenosis with compression of the cauda equina nerve roots and associated edema. The patient underwent surgical excision, and histopathology confirmed osteochondroma, with significant postoperative symptom relief. This case highlights the importance of recognizing spinal osteochondroma as a rare cause of lumbar radiculopathy and underscores the role of cross-sectional imaging in accurate diagnosis and surgical planning.

## Introduction

Osteochondroma is the most common benign bone tumor, typically arising from the metaphysis of long bones. However, spinal involvement is rare, accounting for less than 5% of all osteochondromas [1,2]. When present in the spine, these lesions most commonly arise from the posterior elements, including the lamina, pedicles, and articular processes [3]. Although often asymptomatic, spinal osteochondromas can result in neurological compromise due to spinal canal or foraminal encroachment [4]. Early recognition is essential, as surgical excision is usually curative and prevents permanent neurological deficits.

## Case presentation

A 39-year-old male presented with progressive left-sided lower limb pain for over 3 months. The pain radiated from the lower back to the anterior thigh and medial leg, corresponding predominantly to the L3–L4 dermatomal distribution. It was sharp, severe in intensity, aggravated by movement, and refractory to analgesics and physiotherapy.

There was no history of trauma, prior spinal surgery, fever, weight loss, or known hereditary skeletal disorders such as multiple hereditary exostoses. Family history was non-contributory.

On physical examination, there was localized tenderness over the lumbar spine. Neurological examination revealed reduced motor strength (4/5) in left hip flexion and knee extension, with diminished patellar reflex. Sensory examination showed decreased sensation in the L3–L4 dermatomes on the left side. No bowel or bladder dysfunction was noted.

Laboratory investigations, including complete blood count, erythrocyte sedimentation rate, and C-reactive protein, were within normal limits.

Magnetic resonance imaging (MRI) of the lumbosacral spine demonstrated a well-defined bony lesion arising from the inferior articular process of the L3 vertebra, projecting into the spinal canal. The lesion caused severe canal stenosis with compression of the cauda equina nerve roots and associated T2 hyperintensity suggestive of nerve root edema (Fig. 1A and 1B).

**Fig. 1:**
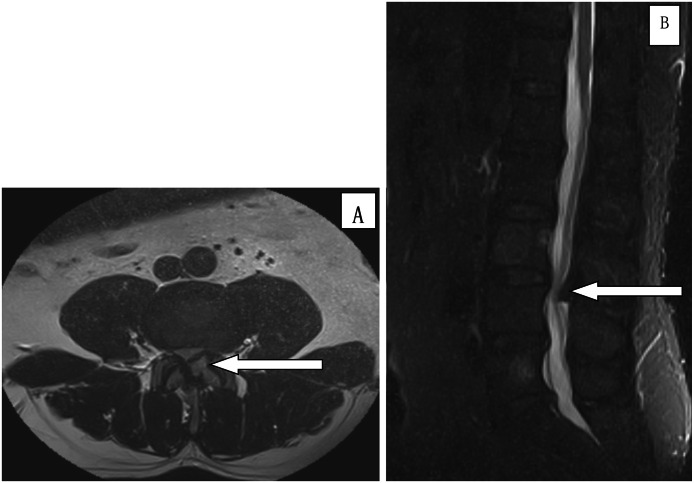
(**A**) T1 axial and (B) STIR sagittal lumbar MRI, White arrows indicate a bony outgrowth compression of the cauda equina nerve roots with associated T2 heterogeneous hyperintensity suggesting edema.

Subsequent computed tomography (CT) confirmed a pedunculated osseous lesion with cortical and medullary continuity with the parent vertebra, consistent with osteochondroma (Figure 2A and 2B), which is considered pathognomonic [5].

**Fig. 2:**
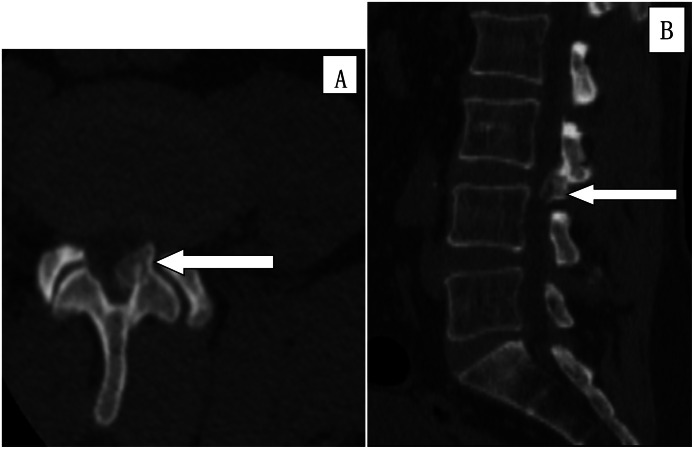
**(A)** Axial and (B) sagittal lumbar CT images, White arrows indicate a well-defined osseous lesion showing cortical and medullary continuity with the parent vertebra, consistent with osteochondroma.

The patient underwent posterior decompression and complete surgical excision of the lesion. Intraoperatively, a bony mass compressing the Dural sac and nerve roots was identified and excised.

Histopathological examination confirmed osteochondroma, demonstrating a cartilage cap overlying mature trabecular bone.

Postoperatively, the patient experienced significant improvement in radicular pain and gradual recovery of motor function during follow-up.

## Discussion

Spinal osteochondromas are rare, with lumbar involvement being less common compared to cervical and thoracic regions [2,4]. These lesions typically arise from secondary ossification centers and therefore predominantly involve the posterior elements of the vertebrae [3].

This case is unique due to the lesion originating from the inferior articular process of L3, an uncommon site, and causing severe radiculopathy through compression of the cauda equina nerve roots. At the L3 level, the spinal cord has already terminated, making cauda equina compression anatomically consistent. The presence of T2 hyperintensity indicating nerve root edema further reflects the severity and chronicity of compression.

Clinical manifestations of spinal osteochondromas depend on lesion location and size. Patients may present with radiculopathy, myelopathy, or neurogenic claudication [6]. Due to their slow growth, symptoms may be insidious and lead to delayed diagnosis.

Imaging plays a crucial role in diagnosis. CT is the modality of choice for identifying cortical and medullary continuity between the lesion and parent bone, which is diagnostic of osteochondroma [5]. MRI is essential for evaluating neural compression and detecting associated signal changes, including edema, as well as assessing cartilage cap thickness, particularly when malignant transformation is suspected [7].

Although malignant transformation is rare in solitary osteochondromas, it should be considered in cases demonstrating rapid growth, pain progression, or a thickened cartilage cap [8]. Recent literature continues to emphasize the importance of imaging in distinguishing benign from malignant lesions and guiding surgical planning [9,10].

Compared with previously reported cases, this case underscores that even relatively small lesions arising from posterior elements can produce significant neurological compromise when projecting into the spinal canal. Early surgical intervention is therefore critical.

Complete surgical excision remains the treatment of choice and is typically curative, with excellent prognosis when performed before irreversible neurological damage occurs [6].

## Conclusion

Posterior element lumbar osteochondroma is a rare but significant cause of radiculopathy due to cauda equina nerve root compression. Recognition of characteristic imaging features, particularly cortical and medullary continuity on CT and neural compression on MRI, is essential for accurate diagnosis. Prompt surgical excision results in favorable clinical outcomes and prevents permanent neurological deficits.

## Patient consent

Both oral and written informed consent were obtained from the patient for publication of this case report and the associated images.

## References

[1] Dahlin D.C., Unni K.K. (1996). Bone tumors: general aspects and data on 8,542 cases.

[2] Albrecht S., Crutchfield J.S., SeGall G.K. (1992). On spinal osteochondromas. J Neurosurg.

[3] Lotfinia I., Vahedi P., Tubbs R.S., Ghavame M., Meshkini A., Mortazavi M.M. (2010). Spinal osteochondroma: review of cases and literature. J Neurosurg Spine.

[4] Gille O., Pointillart V., Vital J.M. (2005). Course of spinal solitary osteochondromas. Spine.

[5] Murphey M.D., Choi J.J., Kransdorf M.J., Flemming D.J., Gannon F.H. (2000). Imaging of osteochondroma: variants and complications. Radiographics.

[6] Xu R., McGirt M.J., Parker S.L., Bydon A., Olivi A., Wolinsky J.P. (2008). Spinal osteochondromas: clinical features and surgical outcomes. Neurosurg Focus.

[7] Yochum T.R., Rowe L.J. (2005). Essentials of skeletal radiology.

[8] Ahmed A.R., Tan T.S., Unni K.K., Collins M.S., Wenger D.E., Sim F.H. (2003). Secondary chondrosarcoma in osteochondroma. J Bone Joint Surg Am.

[9] Kitsoulis P., Galani V., Stefanaki K., Paraskevas G., Karatzias G., Agnantis N.J. (2008). Osteochondromas: review of the clinical, imaging and pathological features. In Vivo.

[10] Czajka C.M., DiCaprio M.R. (2015). What is the proportion of patients with multiple hereditary exostoses who undergo malignant degeneration?. Clin Orthop Relat Res.

